# Post‐Traumatic Growth in Adult Cancer Survivors: A Scoping Review of Psychological Factors, Predictors, and Interventions

**DOI:** 10.1002/ijop.70097

**Published:** 2025-08-28

**Authors:** Anna Nisiraiou, Antonios Bozas, Dimitrios Kyrou, Konstantina Stavrogianni, Maria Vasilopoulou, Georgios ‐ Marios Kalomoiris, Natalia Tsintsifa, Katerina Nikitara, George Koulierakis, Christina Karamanidou

**Affiliations:** ^1^ Institute of Applied Biosciences, Centre for Research & Technology‐Hellas Thessaloniki Greece; ^2^ Aristotle University of Thessaloniki Thessaloniki Greece; ^3^ Hellenic Cancer Federation Athens Greece; ^4^ Department of Public Health Policy University of West Attica Athens Greece

**Keywords:** cancer patients, post‐traumatic growth, scoping review

## Abstract

Post‐traumatic growth (PTG), defined as positive psychological changes following trauma, has garnered attention in recent years within the context of cancer. This scoping review aims to synthesise and map PTG‐related studies published in the last 5 years among adult cancer populations. A comprehensive literature search identified 109 eligible studies published between 2018 and 2023, predominantly cross‐sectional in design, focusing on various cancer types, with a significant proportion examining breast cancer. The findings reveal that PTG is consistently associated with cognitive, emotional, social, and health‐related factors. Resilience, adaptive coping strategies (e.g., positive reappraisal, deliberate rumination, meaning‐making), and social support emerged as robust positive correlates. Conversely, psychological distress (depression, anxiety, intrusive rumination) and poor physical health were generally inversely associated with PTG. Longitudinal studies, while fewer, indicated that coping strategies and social support predict PTG trajectories; PTG can, in turn, influence subsequent adaptive coping and well‐being. Thirteen interventional studies were identified, with six demonstrating significant improvements in PTG in intervention groups, notably those incorporating elements like acceptance, self‐compassion, deliberate rumination, meaning‐making, and social support. This review underscores the complex nature of PTG, highlighting key psychosocial factors that facilitate its development in cancer survivors and suggesting promising avenues for therapeutic interventions.

## 1 Introduction

According to WHO, cancer has become one of the biggest causes of mortality (Stewart and Wild 2014). A cancer diagnosis and treatment are often followed by associated debilitating physical symptoms but also psychological and social difficulties (Costa et al. 2016). Emotional outcomes that have been observed include anxiety, depression, worry, fear, anger, guilt, and general distress (Akechi et al. 2023; Dinapoli et al. 2021; Mehnert et al. 2018). For some patients, the cancer experience is decisively traumatic, with psychological consequences that might result in Post‐Traumatic Stress Disorder (PTSD) (Carletto et al. 2019). Experiencing cancer, with its array of physical, emotional, and existential challenges, may induce a reassessment of an individual's life and values, yielding diverse outcomes ranging from unresolved suffering to more positive outcomes (Vehling and Kissane 2018; Jewett et al. 2022). Indeed, for some individuals, the adversity of a traumatic event such as cancer may ultimately catalyse positive personal growth, representing a potentially desirable byproduct of the cancer experience.

These experiences have given rise to a concept known as posttraumatic growth (PTG) (Tedeschi and Calhoun 1996). PTG refers to the positive psychological changes that individuals can undergo in response to trauma. A common measure of PTG, namely the Posttraumatic Growth Inventory (PTGI), encompasses five dimensions of possible positive psychological reactions to the illness, including new possibilities, relating to others, spiritual change, personal strength, and appreciation of life. Studies indicate a 20.5% prevalence of moderate‐to‐high PTG (among oncology patients), regardless of age or sex (Liu, Thong, et al. 2021). In the past 5 years, there has been a growing interest in exploring the PTG of individuals who have faced highly stressful events, particularly within the context of adult cancer populations.

Positive factors such as healthier habits, better health‐related quality of life and personality traits, such as optimism, have been linked to PTG in cancer patients (Evans et al. 2022; Kolokotroni et al. 2014; Liu, Doege, et al. 2020). Various factors like time since diagnosis (Hamdan et al. 2022) and social support (Gu et al. 2023) have emerged as predictors. However, the understanding of psychological factors and the internal processes associated with PTG is still evolving due to the overlap of these constructs, that is, well‐being and the meaning of life, particularly in how they are operationalised in specific measurement tools (Holtmaat et al. 2019). Notably, therapeutic interventions have demonstrated the potential to foster PTG (Evans et al. 2022; Kissane et al. 2019; Wang, Lin, et al. 2022; Zhu et al. 2022). Recent research highlights promising areas for enhancing patients' PTG, particularly through psychosocial interventions such as mindfulness‐based interventions, positive psychology interventions or by targeting psychological resources such as hope, optimism, spirituality (Casellas‐Grau et al. 2017; Vrontaras et al. 2023). Moreover, several studies have examined factors that mediate and/or moderate PTG, including resilience, social support, and cognitive processing (Casellas‐Grau et al. 2017; Gori et al. 2021; Li et al. 2020).

Nevertheless, researchers have underscored the challenge of elucidating PTG due to its similarity with other positive constructs, such as resilience and meaning‐making (Casellas‐Grau et al. 2017; Holtmaat et al. 2019). Unlike resilience, which refers to the ability to maintain or quickly regain psychological functioning in the face of adversity, PTG involves a transformative process that results in perceived psychological growth beyond the individual's pre‐trauma baseline. Similarly, other positive constructs such as meaning‐making typically capture short‐term positive reinterpretations of adversity, whereas PTG encompasses more profound and potentially lasting changes in identity, life attitude, and interpersonal relationships (Tedeschi and Calhoun 2004). Moreover, there is a predominant focus on the negative aspects of cancer within the existing body of cancer research. Consequently, meta‐analyses conclude that the current state of research remains inconclusive in terms of pinpointing clinical predictors, correlates, and mediators of PTG (Casellas‐Grau et al. 2017). However, considering the growing body of research on PTG and the evidence that suggests the possible importance that PTG may have in cancer treatment and survivorship from well‐being to health‐related behaviours, it is crucial to delve into the factors that facilitate and promote PTG, providing a comprehensive understanding of its dynamics. This knowledge can inform more targeted and effective approaches to supporting cancer patients and survivors through a construct of unique clinical relevance (Vrontaras et al. 2023).

### 1.1 Aim

Against this background, the present scoping review aims to identify and map the existing PTG literature by including cross‐sectional studies, longitudinal studies, and interventions among adults with cancer. The main research questions are the following:

- What is the range of studies mapping PTG in the adult cancer population over the last 5 years?
- What are the key factors associated with PTG?
- What outcomes are relevant to PTG?

The PRISMA Statement modified for scoping reviews was followed (Tricco et al. 2018). The methodology includes identification of the research question, identification of relevant studies, study selection, charting of the data, and summarising and reporting the results (Tricco et al. 2018). No ethical permission was required.

## 2 Methodology

### 2.1 Search Strategy

Electronic literature searches were performed using PsycNet, PubMed, Scopus, Cochrane Library, and ProQuest databases. Publications covering the last 5 years were included. All references were imported into the Zotero Electronic Database for further management.

English language literature was searched from 2018 to 2023 to obtain recent data using the keywords “cancer,” “Post Traumatic Growth,” “PTG,” and/or “adults” from studies written in English.

### 2.2 Inclusion Criteria

(i) studies referring to posttraumatic growth (PTG), including cross‐sectional, longitudinal, or interventional research designs; (ii) focused on adult cancer patients across various cancer types; (iii) employed the Posttraumatic Growth Inventory (PTGI), including its extended (PTGI‐X) or short‐form (PTGI‐SF) versions (Tedeschi and Calhoun 1996); (iv) had a sample size of at least 100 participants; (v) were peer‐reviewed journal articles published in the last 5 years; (vi) were written in English.

### 2.3 Exclusion Criteria

(i) Reviews, editorials, letters, book chapters and case reports; (ii) a different PTGI measure; (iii) paediatric population; (iv) exclusive qualitative methodology; (v) non‐cancer population; (vi) mixed study population not assessed separately; (vii) different language (not English) (viii) published above the last 5 years.

The initial findings were inputted into the Rayyan software for additional organisation. Duplicate studies were verified for precision. In the initial screening stage, two reviewers independently evaluated all titles and abstracts. In the following round, both reviewers scrutinised each full‐text article and extracted relevant details like study design, inclusion criteria, sample characteristics, cancer‐related variables, group comparisons, intervention types, and PTGI outcomes. Publications that did not meet inclusion criteria were excluded. Next, the researchers individually read the full text of eligible articles and evaluated the eligibility of each article. Finally, eligible studies were chosen through a cross‐checking method in a research team meeting. Any inconsistencies were resolved by a third reviewer when needed. A standardised Excel form was employed for the review and data extraction from the articles.

## 3 Results

### 3.1 Search Outcomes

**FIGURE 1:**
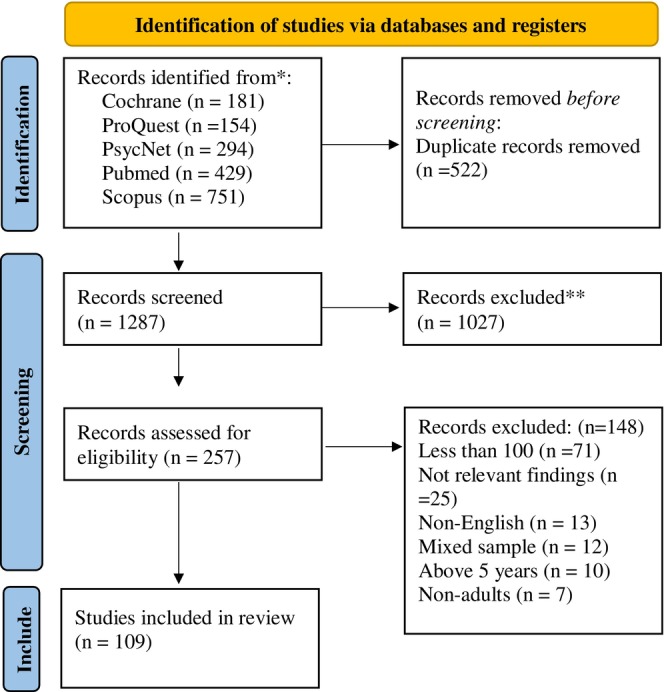
PRISMA diagram showing the identification, screening and inclusion of articles for scoping.

A total of 1812 titles and abstracts were identified and assessed for eligibility. After removing duplicates, the 1287 titles were retained. Following the screening of all full‐text articles, the final number of eligible studies was 109 (Figure 1).

### 3.2 Data Items

The information extracted from the articles encompassed details on methodology (study type, country, repeated measures time points), participants (number of participants in each group, type of cancer, gender, age), intervention (type, group or individual setting), and results (effect size, statistical significance).

The present review included the reporting of PTGI scores. PTGI is a self‐reported assessment tool with a total score that ranges from 0 to 105, where higher scores reflect greater PTG (Tedeschi and Calhoun 1996).

### 3.3 Sample Characteristics

48.942 patients with cancer or survivors participated in the 109 studies (M: 437, SD: 976). Across articles that reported mean participant age, the grand mean for age was 54.20 (SD: 7.59) years and the median was 54.38 years. In studies that reported participants gender, 14.993 participants (31% of the total participant number) were male, and 28.265 participants (58% of the total participant number) were female.

**FIGURE 2:**
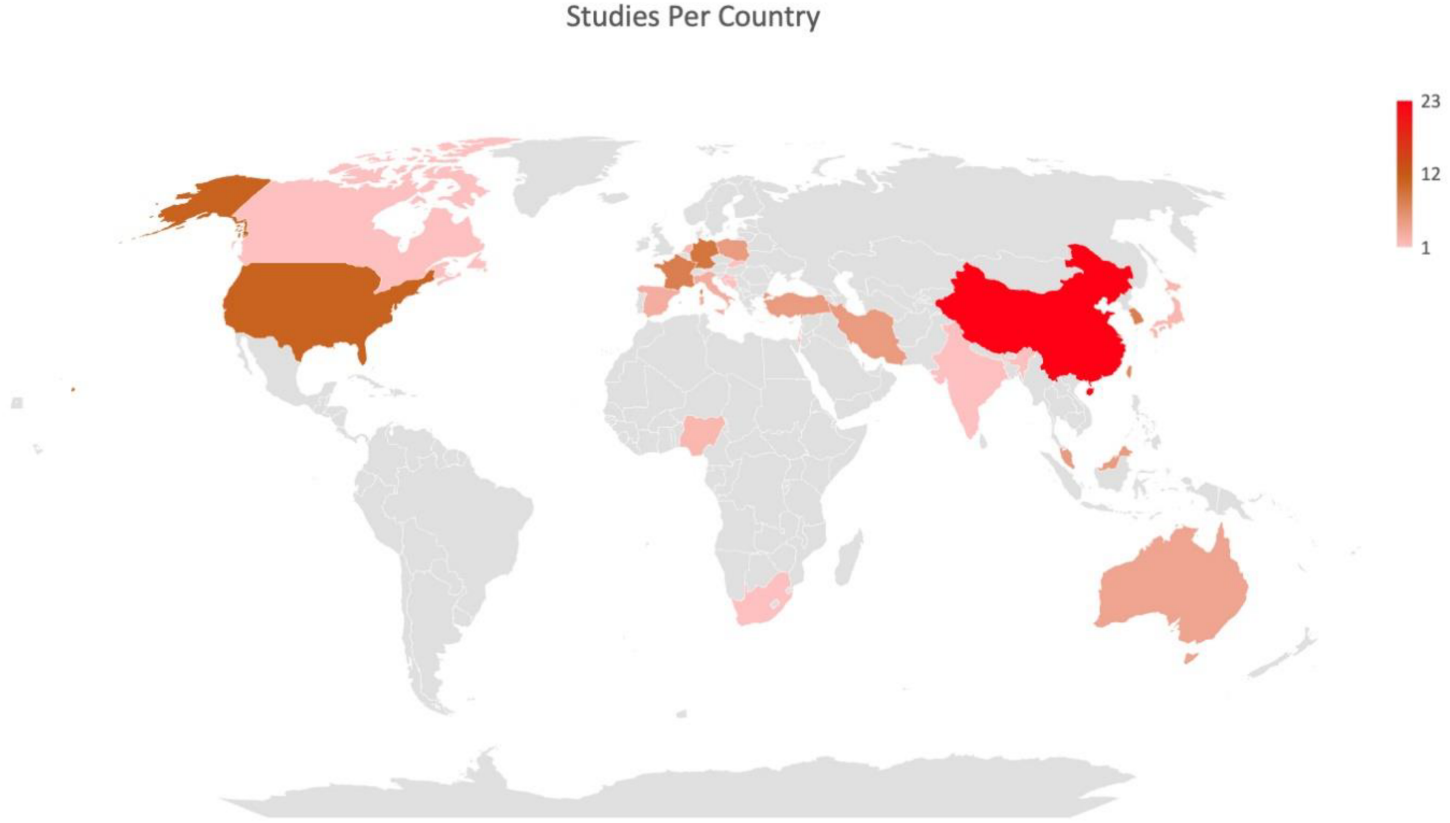
This map *illustrates* the distribution of studies conducted across different countries. The varying shades of red represent the number of studies, with darker shades indicating a higher number of studies.

Twenty‐three studies were originated from China (Gu et al. 2023; Han et al. 2021; Jie et al. 2020; Li et al. 2016, 2020; Li 2022; Lianchao and Tingting 2020; Liu, Doege, et al. 2020; Peng et al. 2019; Shi et al. 2021, 2022; Song et al. 2021, 2022; Suo et al. 2021; Tu et al. 2021; Wang, Li, et al. 2022; Wang, Lin, et al. 2022; Zhang et al. 2019, 2020, 2021; Zhou et al. 2021, 2023; Zhu et al. 2022), 11 from the USA (Applebaum et al. 2021; Chu et al. 2022; Hlubocky et al. 2022; Jewett et al. 2022; Mell et al. 2022; Messelt et al. 2021; Rider Mundey et al. 2019; Schwartz et al. 2022; Senger et al. 2024; Sheikh‐Wu et al. 2022; Wong et al. 2019); nine studies were from Germany; however, it is important to note that the studies by Blickle et al. (2024) and Onyedibe et al. (2024) were based on the same sample (Aderhold et al. 2019; Blickle et al. 2024; Ernst et al. 2023; Liu, Doege, et al. 2021; Liu, Thong, et al. 2021; Liu et al. 2023; Onyedibe et al. 2024; Philipp et al. 2020; Scherer‐Trame et al. 2022); eight from France (Bourdon et al. 2019; Corman et al. 2021; Couderc et al. 2023; Dubuy et al. 2022; Evans et al. 2022; Porro et al. 2022; Rey et al. 2021; Rezaee Vessal et al. 2022) and the Republic of Korea (Choi et al. 2023; Kim and Son 2021; Kim and Shin 2022; Kim et al. 2021; Oh et al. 2021; Yang and Ha 2019; Yi et al. 2023; Yun et al. 2020); seven from Taiwan (Chang et al. 2022; Chen et al. 2019; Longcoy et al. 2023; Tu 2022; Tu et al. 2020; Wang et al. 2023; Yang et al. 2023); five from Iran (Heidarzadeh et al. 2021; Honari et al. 2022; Karimzadeh et al. 2021; Moghadam et al. 2021; MoshirPanahi et al. 2020); Poland (Houn et al. 2022; Michalczyk et al. 2022; Rzeszutek et al. 2020; Szcześniak et al. 2022; Trzmielewska et al. 2019); Malaysia (Hamdan et al. 2022; Leong Abdullah et al. 2019; Nik Jaafar et al. 2021; Nik Jaafar, Abd Hamid, et al. 2022; Nik Jaafar, Hamdan, et al. 2022) and Turkey (Aydin and Kabukçuoğlu 2020; Gundogmus et al. 2022; Boyacıoğlu et al. 2022; Gür and Öztürk 2023; Özönder Ünal et al. 2023); four from Australia (Dyer et al. 2016; Kissane et al. 2019, 2023; McErlean et al. 2023); three from Italy (Gori et al. 2021; Romeo et al. 2019, 2020) and Spain (Guil et al. 2022; Lleras de Frutos et al. 2020; Ochoa‐Arnedo et al. 2021); two from the Netherlands (Holtmaat et al. 2019, 2020) and Nigeria (Aliche 2023; Aliche et al. 2023); and one from Bosnia and Herzegovina (Lisica et al. 2019), Canada (Daniel et al. 2021), Croatia (Mostarac and Brajković 2022), India (Thakur et al. 2022), Israel (Hamama‐Raz et al. 2019), Japan (Akechi et al. 2023; Matsui and Taku 2023), South Africa (Ofei et al. 2023) and Slovakia (Baník et al. 2022) (for an overview see Figure 2).

The majority of studies reviewed (*n* = 74) were cross‐sectional (Aderhold et al. 2019; Aliche 2023; Aliche et al. 2023; Applebaum et al. 2021; Aydin and Kabukçuoğlu 2020; Gundogmus et al. 2022; Baník et al. 2022; Boyacıoğlu et al. 2022; Chang et al. 2022; Choi et al. 2023; Daniel et al. 2021; Dubuy et al. 2022; Dyer et al. 2016; Ernst et al. 2023; Gori et al. 2021; Gu et al. 2023; Guil et al. 2022; Gür and Öztürk 2023; Heidarzadeh et al. 2021; Hlubocky et al. 2022; Holtmaat et al. 2019; Honari et al. 2022; Chen et al. 2019; Houn et al. 2022; Jewett et al. 2022; Karimzadeh et al. 2021; Kim and Son 2021; Kim and Shin 2022; Kim et al. 2021; Leong Abdullah et al. 2019; Li et al. 2016, 2020; Li 2022; Lianchao and Tingting 2020; Lisica et al. 2019; Liu, Zhang, et al. 2020; Liu, Doege, et al. 2021; Liu, Thong, et al. 2021; Longcoy et al. 2023; McErlean et al. 2023; Messelt et al. 2021; Michalczyk et al. 2022; Moghadam et al. 2021; MoshirPanahi et al. 2020; Mostarac and Brajković 2022; Nik Jaafar, Abd Hamid, et al. 2022; Ofei et al. 2023; Oh et al. 2021; Özönder Ünal et al. 2023; Peng et al. 2019; Porro et al. 2022; Rezaee Vessal et al. 2022; Rider Mundey et al. 2019; Romeo et al. 2019; Rzeszutek et al. 2020; Sheikh‐Wu et al. 2022; Shi et al. 2021, 2022; Song et al. 2021, 2022; Suo et al. 2021; Szcześniak et al. 2022; Thakur et al. 2022; Tu et al. 2020; Tu 2022; Wong et al. 2019; Yang et al. 2023; Yang and Ha 2019; Yi et al. 2023; Zhang et al. 2019, 2020, 2021; Zhou et al. 2021, 2023). Of the remaining studies, 21 were longitudinal (Blickle et al. 2024; Bourdon et al. 2019; Corman et al. 2021; Couderc et al. 2023; Evans et al. 2022; Hamama‐Raz et al. 2019; Hamdan et al. 2022; Jie et al. 2020; Liu et al. 2023; Matsui and Taku 2023; Mell et al. 2022; Nik Jaafar et al. 2021; Nik Jaafar, Abd Hamid, et al. 2022; Onyedibe et al. 2024; Philipp et al. 2020; Rey et al. 2021; Romeo et al. 2020; Scherer‐Trame et al. 2022; Schwartz et al. 2022; Senger et al. 2024; Wang et al. 2023). It should be noted that Liu, Doege, et al. (2021); Liu, Thong, et al. (2021); Liu et al. (2023) drew on the same participant sample across these publications. One used a mixed‐methods, qualitative and quantitative (cross‐sectional) design (Trzmielewska et al. 2019) and 13 were interventional (Akechi et al. 2023; Chu et al. 2022; Han et al. 2021; Holtmaat et al. 2020; Kissane et al. 2019, 2023; Lleras de Frutos et al. 2020; Ochoa‐Arnedo et al. 2021; Tu et al. 2021; Wang, Li, et al. 2022; Wang, Lin, et al. 2022; Yun et al. 2020; Zhu et al. 2022).

Regarding the type of cancer, 46 studies included participants with mixed types (Aderhold et al. 2019; Aliche 2023; Aliche et al. 2023; Applebaum et al. 2021; Aydin and Kabukçuoğlu 2020; Baník et al. 2022; Blickle et al. 2024; Bourdon et al. 2019; Choi et al. 2023; Daniel et al. 2021; Dyer et al. 2016; Evans et al. 2022; Gori et al. 2021; Gür and Öztürk 2023; Han et al. 2021; Heidarzadeh et al. 2021; Holtmaat et al. 2019, 2020; Jewett et al. 2022; Kissane et al. 2019, 2023; Leong Abdullah et al. 2019; Li et al. 2016; Lianchao and Tingting 2020; Liu, Doege, et al. 2021; Liu, Thong, et al. 2021; Liu et al. 2023; Lleras de Frutos et al. 2020; Matsui and Taku 2023; McErlean et al. 2023; MoshirPanahi et al. 2020; Mostarac and Brajković 2022; Ochoa‐Arnedo et al. 2021; Onyedibe et al. 2024; Özönder Ünal et al. 2023; Philipp et al. 2020; Rezaee Vessal et al. 2022; Rider Mundey et al. 2019; Schwartz et al. 2022; Szcześniak et al. 2022; Trzmielewska et al. 2019; Wang, Lin, et al. 2022; Yang et al. 2023; Yang and Ha 2019; Yun et al. 2020; Zhang et al. 2020), 34 studies included solely breast cancer participants (Akechi et al. 2023; Gundogmus et al. 2022; Chu et al. 2022; Dubuy et al. 2022; Gu et al. 2023; Guil et al. 2022; Hamama‐Raz et al. 2019; Honari et al. 2022; Chen et al. 2019; Karimzadeh et al. 2021; Li et al. 2020; Li 2022; Lisica et al. 2019; Liu, Doege, et al. 2020; Longcoy et al. 2023; Michalczyk et al. 2022; Moghadam et al. 2021; Ofei et al. 2023; Porro et al. 2022; Rey et al. 2021; Romeo et al. 2019, 2020; Senger et al. 2024; Shi et al. 2021, 2022; Suo et al. 2021; Thakur et al. 2022; Tu et al. 2020; Tu 2022; Wang, Li, et al. 2022; Wang et al. 2023; Wong et al. 2019; Yi et al. 2023; Zhu et al. 2022), eight studies with gynaecological cancer (Hlubocky et al. 2022; Mell et al. 2022; Messelt et al. 2021; Oh et al. 2021; Song et al. 2021, 2022; Zhou et al. 2021, 2023), five with colorectal cancer (Kim and Son 2021; Kim et al. 2021; Scherer‐Trame et al. 2022; Sheikh‐Wu et al. 2022; Zhang et al. 2019), four with head and neck cancer (Chang et al. 2022; Hamdan et al. 2022; Nik Jaafar et al. 2021; Nik Jaafar, Abd Hamid, et al. 2022; Nik Jaafar, Hamdan, et al. 2022) four with lung cancer (Couderc et al. 2023; Peng et al. 2019; Tu et al. 2021; Zhang et al. 2021), three with haematological cancer (Boyacıoğlu et al. 2022; Corman et al. 2021; Ernst et al. 2023), two with gastrointestinal cancer (Houn et al. 2022; Rzeszutek et al. 2020), and one with brain cancer (Kim and Shin 2022) and hepatocellular carcinoma (Jie et al. 2020).

### 3.4 Cross‐Sectional Associations

Seventy‐four studies identified various factors associated, either positively or negatively, with PTG (Table 1). Assessment measures commonly used among the studies were the Connor‐Davidson Resilience Scale (CD‐RISC) (Choi et al. 2023; Gori et al. 2021; Gu et al. 2023; Li et al. 2020; Zhang et al. 2019), Event‐Related Rumination Inventory (ERRI) (Gür and Öztürk 2023; Lianchao and Tingting 2020; Rider Mundey et al. 2019; Song et al. 2021; Szcześniak et al. 2022), Medical Coping Modes Questionnaire (MCMQ) (Gu et al. 2023; Zhang et al. 2021; Zhou et al. 2021), Multidimensional Scale of Perceived Social Support (MSPSS) (Kim et al. 2021; Romeo et al. 2019; Zhou et al. 2023), Meaning in Life Questionnaire (MLQ) (Aliche et al. 2023; Moghadam et al. 2021; Mostarac and Brajković 2022), The Mindful Attention Awareness Scale (MAAS) (Aliche 2023; Aliche et al. 2023; Lianchao and Tingting 2020), the Hospital Anxiety and Depression Scale (HADS) (Aderhold et al. 2019; Li et al. 2016; Peng et al. 2019), the ERRI (Gür and Öztürk 2023; Lianchao and Tingting 2020; Szcześniak et al. 2022) and the MCMQ (Zhang et al. 2021; Zhou et al. 2021).

**TABLE 1:** Cross sectional studies on PTG.

References	Country of study	*n*	Gender female/male of total %	Population mean Age (SD) control/intervention	Type of cancer	Associations
Aderhold et al. (2019)	Germany	157	F: 53.5% M: 46.5%	M: 58 SD: 14.1	Mixed	Depressive symptoms (HADS) (−)
Aliche (2023)	Nigeria	550 patients	F: 53.1% M: 46.9%	M: 38.51 SD: 6.73	Mixed	Mindfulness (MAAS) (+)—Positive reappraisal (CERQ) (+)—Self‐Compassion (SCS‐SF) (+)
Aliche et al. (2023)	Nigeria	957 cancer patients	F: 53.2 M: 46%	M: 38.54 SD: 66.71	Mixed	Mindfulness (MAAS) (+)—Meaning of life (MLQ) (+)
Applebaum et al. (2021)	USA	209	F: 32% M: 68%	M: 57.6 SD: 11.3	Mixed	Significant high correlation between BFS and PTGI Meaning: Both questionnaires assess the same underlying construct of positive psychological change, as high correlation between the scales has been observed
Aydin et a. (2020)	Turkey	265	F: 49.1% M: 50.09%	M: 54.82 SD: 12.52	Mixed	Psychometric properties and Factor Structure‐ Turkey
Gundogmus et al. (2022)	Turkey	71	F: 100%	M: 50.52 SD: 9.04	Breast	Resilience (+) Depression (−) (in some groups) Inflammation (hsCRP) (−)
Baník et al. (2022)	Slovakia	126	F: 79% M: 21%	M: 64 SD: 8.74	Mixed	Posttraumatic stress symptoms (PTSS) (U) PTSS demonstrates a curvilinear correlation with PTG, under which both high and low levels of PTSS are related with low levels of PTG. Contrary, when PTSS is moderate, PTG is reported to be at a higher level.
Boyacıoğlu et al. (2022)	Turkey	111	F: 44.1% M: 55.9%	M: 50.45 SD: 16.03	Haematological	Negative Religious Coping (RCS) (+) Participants with higher negative religious coping (e.g., Feeling punished or abandoned by God) is positively associated with PTG—Age (−)
Chang et al. (2022)	Taiwan	114	F: 7.9 M: 92.1	M: 54.59 SD: 1.06	Head and Neck	Fear of Progression (FCR) (+)—Anxiety (SAI) (−)—Having cancer recurrence (+)—Higher level of education (+)—Longer time since Onset Event, e.g., diagnosis (+)
Chen et al. (2019)	Taiwan	145	F: 100%	M: 52.25 SD: 10.45	Breast	– Post‐traumatic stress symptoms (SPAN‐C) (+) – Family support (APGAR) (+) – Receiving chemotherapy (+) – Years of education (+) – Anxiety symptoms (HADS—A) (+)
Choi et al. (2023)	Republic of Korea	164	F: 100%	M: 55.2	Mixed	– Resilience (CD‐ RISC) (+) – Problem‐Solving Focused Coping (CSI) (+)
Daniel et al. (2021)	Canada	463	F: 88%	M: 30.28 SD: 4.68	Mixed	– Knowledge of fertility risk (+) – Not engaging in fertility preservation because of their own choice or doctor's recommendation (+)
Dubuy et al. (2022)	France	379			Mixed	Psychometric properties and Factor Structure‐ France
Dyer et al. (2016)	Australia	441	M: 57%	Median: 54 years	Mixed	PGTI was not associated with adherence to cancer screening (0)
Ernst et al. (2023)	Germany	633	F: 44.4%	M: 35 SD: 5.70	Haematological	– Mental distress (PTSD‐7, GAD‐2, JSS) (−) – Depression (PHQ‐9) (−) – Antidepressant use (+) – Resilience (BRCS) (+) – Age (+) – Being in relationship (+) – Having children (+)
Gori et al. (2021)	Italy	154	F: 90% M: 10%	M: 51.4 SD: 11.25	Mixed	– Resilience (I‐CD‐RISC‐10) (+) – Positive attitude (COPE‐NVI) (+) – Openness (I‐TIPI) (−)
Gu et al. (2023)	China	115	F: 100%		Breast	– Social Support (PSSS) (+) – Resilience (CD‐RISC) (+) – Coping style (MCMQ) (+)
Guil et al. (2022)	Spain	636 Breast Cancer Survivors: 56 healthy controls: 580	F: 100%	M: 51.77 SD: 8.92	Breast	– Survivorship (+) – Perceived emotional intelligence (+)
Gür and Öztürk (2023)	Turkey	139	F: 47.5% M: 52.5%	M: 59.31 SD: 11.33	Mixed	– Intrusive rumination (ERRI) (−) – Deliberate rumination (ERRI) (+) – Disruption in Basic Beliefs (Basic Beliefs Inventory) (+)
Heidarzadeh et al. (2021)	Iran	272			Mixed	– Psychometric properties and Factor Structure—Persian
Hlubocky et al. (2022)	USA	174	F: 100%	Median: 59	Ovarian	– Quality of life‐(FACT‐O/HRQOL) (+) – 1Resilience (BRS) (+)
Holtmaat et al. (2019)	Netherlands	170	F: 82% M: 20%	M: 57 SD: 10	Mixed	– Psychological Well‐Being (+) – Personal Meaning profile (+)
Honari et al. (2022)	Iran	136	F: 100%	M: 48.6 SD: 11.3	Breast	– Post‐Traumatic Stress Disorder (PTSD) (0)
Houn et al. (2022)	Poland	190	F: 45.79% M: 54.21%	M: 63.43 SD: 10.89	Gastrointestinal	– Total resource levels (e.g., economic) (COR‐E) (+) – Social support was found to enhance individuals' perceived and actual levels of personal resources—particularly economic and political ones—which in turn facilitated post‐traumatic growth,
Nik Jaafar, Abd Hamid, et al. (2022)	Malaysia	190	F: 45.8% M: 54.2%		Head and Neck	– Fear of Cancer Progression (FoP‐Q‐SF) (−) – Patient's physical and daily living unmet needs (SCNS‐34) (−)
Jewett et al. (2022)	USA	236	F: 100%	M = 61.7 SD = 10.9	Mixed	– Sense of connection with others (three exploratory measures of sense of connections) (+)
Karimzadeh et al. (2021)	Iran	210	F: 100%	M = 47.6 SD = 10.48	Breast	– Satisfaction of basic psychological needs (BPNS‐Scale) (+) – Maladaptive Schema (YSQ‐SF) (+) – Emotion regulation (ERQ) (+)
Kim and Shin (2022)	Republic of Korea	114	F: 36% M: 64%	M: 55.46 SD: 11.2	Brain	– Cancer Coping Questionnaire (+) – Healthcare professionals' support (HPS) (+) – Having a primary caregiver (+)
Kim and Son (2021)	Republic of Korea	140	F: 45.7% M: 54.3%	M: 68.18 SD: 9.95	Colorectal	– Social support (Multidimensional Scale of Perceived Social Support) (+) – Psychological well‐being (City of Hope Quality of Life–Ostomy) (+) – Social well‐being (City of Hope Quality of Life–Ostomy) (+) – Spiritual well‐being (City of Hope Quality of Life–Ostomy) (+)
Kim et al. (2021)	Republic of Korea	143	M: 100%		Rectal	– Sexual function (IIEF) (+) – Social support (MSPSS) (+)
Leong Abdullah et al. (2019)	Malaysia	195	F: 73% M: 27%	M = 53 years SD = 10.25	Mixed	– Hope (HS) (+) – Spousal Support (SSSS) (+) – Female gender (+) – Islamic religious belief (+)
Li et al. (2016)	China	198	F: 47% M: 51.5%	M = 57.70 SD = 12.11	Mixed	– Anxiety (HADS) (−) – Depression (HADS) (−)
Li et al. (2020)	China	244	F: 100%		Breast	– Resilience (CD‐RISC) (+) – Anxiety (HADS) (−) – Depression (HADS) (−)
Li (2022)	China	405	F: 100%	M = 49.80 SD = 9.66	Breast	– Body image (BIS) (−) – Social support (PSSS) (+) – Depressive symptoms (CES‐D) (−)
Lianchao and Tingting (2020)	China	309	F: 38.8% M: 61.2%	M: 58.85 SD: 3.26	Mixed	– Mindfulness (MAAS) (+) – Deliberate Rumination (ERRI) (+) – Intrusive Rumination (ERRI) (−)
Lisica et al. (2019)	Bosnia and Herzegovina	100	F: 100%	M: 55.02 SD: 10.03	Breast	– Self‐Esteem (Scale of Self‐Esteem) (+) – Optimism (Optimism and Pessimism Scale) (+) – Proactive Coping (Scale of Proactive Coping) (+)
Liu, Doege, et al. (2020)	China	612	Not reported	M: 46.8 SD: 13.1	Breast	– Social Support (Furman and Buhrmester Network of Relationships Inventory) (+) – PTSS (PSS) (+) – Anxiety (GAD‐7) (+) – Depression (PHQ‐9) (+)
Liu, Doege, et al. (2021)	Germany	6952	M: 47.2%	M: 61 SD: 8.9	Mixed	– No unresolved/untreated symptoms (Cognitive appraisal of medical care) (+) – Satisfaction with care for other diseases (Cognitive appraisal of medical care) (−) – Time since diagnosis (−) – Cancer Distress (QSC‐R10) (+)
Liu, Thong, et al. (2021)	Germany	6952	F: 53.8% M: 47.2%	M: 69.1 SD: 8.9	Mixed	– Benefit Finding (BFS) (+)
Longcoy et al. (2023)	Taiwan	91	F: 100%	M: 49 SD: 9.07	Breast	– Resilience (Resilience Scale) (+) – QoL (WHOQOL‐BREF) (+)
McErlean et al. (2023)	Australia	441	F: 43.31% M: 56.69%	M: 52.17 SD: 12.64	Allogeneic Haematopoietic Stem Cell Transplantation (HSCT) survivors	– Anxiety, Stress, Depression (DASS21) (+) – Quality of life (FACT‐BMT) (+) – Complementary therapy use (+) – Psychosocial care (+) – Female gender (+) – Younger age (+)
Messelt et al. (2021)	USA	242	F: 100%	Not reported	Mixed	– Endometrial Cancer < (lower PTG) Ovarian Cancer
Michalczyk et al. (2022)	Poland	100	F: 100%	Not reported	Breast cancer	– Resilience (KOP‐26) (+) – Women who stated living with cancer was their only experience of a traumatic event < (lower PTG) women who had experienced many traumatic events
Moghadam et al. (2021)	Iran	213	Not reported	M: 52 SD: 16	Breast	– Deliberate rumination (RRS) (+) – Intrusive rumination (RRS) (−) – Disruption of core beliefs (DBI) (+) – Social support (brief COPE) (+) – Negative religious coping (brief COPE) (−) – Positive religious coping (brief COPE) (+) – Emotion—based coping (CERQ) (+) – Search for meaning (MLQ) (−) – Presence of meaning (MLQ) (+) – Problem‐based coping (brief COPE) (+)
MoshirPanahi et al. (2020)	Iran	167	F: 55.7% M: 44.3%	M: 53.00, SD: 27.57	Mixed	– Memory specificity (AMT) (+) – Positive cognitive processing (+) (CPOTS) – Positive attentional biases (APNIS) (+) – Negative attentional biases (APNIS) (0)
Mostarac and Brajković (2022)	Croatia	149	F: 105 M: 44	M: 49.18	Mixed	– Meaning in Life (MLQ) (+) – Life satisfaction (SWLS) (+)
Ofei et al. (2023)	South Africa		F: 100%		Breast	– Perceived Social Support (PSS) (+) – Religion (DUREL) (+) – Hope (SHS) (+) – Optimism (LOT‐R) (+) – Benefit Finding (BFS) (+)
Oh et al. (2021)	Republic of Korea	148	F: 100&	Not reported	Ovarian	– Religion (+) – Cancer Coping (K‐CCQ) (+) – Posttraumatic stress (IES‐R‐K) (−)
Özönder Ünal et al. (2023)	Turkey	253	M: 37.2% F: 62.8%	M: 57.29 SD: 10.38	Mixed	– Self‐Compassion (SCS) (+) – Mindfulness (FMI) (+) – Acceptance and Action (AAQ‐II) (−) – Cognitive Fusion (CFQ) (−)
Peng et al. (2019)	China	173	F: 31.2% M: 68.8%	Not reported	Lung	– Depression (HADS) (−) – Anxiety (HADS) (−) – Negative Coping (SCSQ) (−) – Active Coping (SCSQ) (+) – Increased time since diagnosis (+)
Porro et al. (2022)	France	239	F: 100%	M: 47.13 SD: 9.71	Breast	– Psychometric properties of 3 short versions of PTG
Rezaee Vessal et al. (2022)	France	338 (152 survirors and 186 general population)	M: 21.7% (cancer) M: 45.2% (general population)	M: 57.3 (cancer)/ M: 41.7 (general population)	General to traumatic even, including cancer survivors	– Resilience (CD‐RISC) (+)
Rider Mundey et al. (2019)	USA	221	F: 74.7% M: 24.9%	Not reported	Mixed	– Deliberate Rumination (ERRI) (+) – Emotional intelligence (EI) (+)
Romeo et al. (2019)	Italy	123	F: 100%	M: 54.3 SD: 8.0	Breast	– Fatalism (Mini‐MAC) (+) – Perceived Social Support (MSPSS) (+)
Rzeszutek et al. (2020)	Poland	925 (190 with gastrointestinal cancer, 355 psoriasis, 380 non clinical)	F: 45.8% M: 54.2%	M: 63.43 SD: 10.89 (for cancer patients)	Gastrointestinal	– Levels of Resources (COR‐E) (+)
Sheikh‐Wu et al. (2022)	USA	*n* = 117	F: 44% M: 66%	M: 55.31 SD: 11.62	Colorectal	– Quality of Life (QLI) (+)
Shi et al. (2021)	China	*N* = 133	F: 100%	Not reported	Breast	– Dyadic Coping (C‐DCI) (+) – Intimate relationship (MAT) (−)
Shi et al. (2022)	China	*N* = 789	F: 100%	M: 55	Breast	– Resilience (+) (RS‐14) – Recovery (+) (QPR) – “Impact on daily life due to COVID‐19,” (descriptive) (+)
Song et al. (2021)	China	*N* = 400	F: 100%	M: 47.3 SD: 10.4	Gynaecological	Measuring PTG of Cancer Patient Spouses
Song et al. (2022)	China	Patients (*N* = 400)/Spouses (*N* = 400)	F = 50% M = 50%	Not reported	Gynaecological Cancer couples	– Deliberate Rumination (ERRI) (+) – Intrusive Rumination (ERR) (−) – Self—Disclosure (DDI) (−) – Spouse's Self Disclosure (DDI) (+)
Suo et al. (2021)	China	*N* = 206 mixed (patients and spouses)		M: 45.7 SD: 8.78 (for cancer patients)	Breast	– Positive self – perceived Dyadic Coping – Marital Adjudgment (MAS) (+) – Positive Dyadic Coping (spouse) (DCI) (+) – Marital Adjudgment (spouse) (MAS) (+)
Szcześniak et al. (2022)	Poland	*N* = 215	F: 92.60% M: 7.40%	M: 37.74 SD: 13.97	Mixed	– Word Assumptions WAS (Benevolence of the worlds, Meaningfulness, and Worthiness of the self) (+) – Intrusive rumination (ERRI) (−) – Deliberate rumination (ERRI) (+)
Thakur et al. (2022)	India	*N* = 700	F: 100%	M: 43.25 SD: 8.53	Breast	– Depression, Anxiety, and Stress (DASS‐21) (−) – Treatment completion time (+) – Body Image (Body Image Scale) (0)
Tu et al. (2020)	Taiwan	201	F: 100%	M: 51.54 SD: 9.7	Breast	– Resilience (CD‐RISC) (+) – Negative‐Affect coping (Mini—MAC) (0) – Positive‐Acceptance (Mini—MAC) (+)
Tu (2022)	Taiwan	*N* = 201	F: 100%	M: 51.54 years SD: 9.7	Breast	– Resillience (CD‐RISC) (+) – Intrusion and Brooding Rumination (MRIS) (−) – Instrumentality (MRIS) (+)
Wong et al. (2019)	USA	*N* = 136	F: 100%	M: 57.75 SD: 9.22	Breast cancer	– Self‐Stigma (Self‐Stigma Scale) (−) – Quality of life (FACT‐B) (+)
Yang and Ha (2019)	Republic of Korea	*N* = 121	F: 64.5% M: 35.5%	M: 67.63 SD: 9.35	Mixed	– Social/family well‐being–(FACT‐G) (+) – Functional wellbeing—(FACT‐G) (+)
Yang et al. (2023)	Taiwan	*N* = 141	F: 58.16% M: 41.84%	M: 56.4 SD: 10.2	Mixed	– Spirituality (Spiritual Health Scale‐Short Form) (+) – Post Traumatic Stress (Posttraumatic Stress Reaction Index‐Short Form) (0)
Yi et al. (2023)	Republic of Korea	*N* = 143	F: 100%	M: 52.47 SD: 8.23	Breast	– Successful Aging (SAI) (+)
Zhang et al. (2019)	China	*N* = 157	M: 58.6% F: 41.4%		Colorectal	– Self‐Perceived Burden Scale (SPBS) (−) – Resilience (CD‐RISC) (+) – Retirement (+) – Affordability for medical expenses – Shorter duration of illness (+)
Zhang et al. (2021)	China	*N* = 540	M: 71.2% F: 28.8%	Not reported	Lung	– Social support (SSR) (+) – Cognitive reappraisal (ERQ)+ – Internal locus of control (IPC) (+) – Avoidance coping (MCMQ) (+) – Acceptance‐resignation coping (MCMQ) (−)
Zhang et al. (2020)	China	*N* = 1221	M: 9.1% F: 90.1%	M: 61.77 SD: 8.58	Mixed	– Positive coping strategies (SSSQ) (+) – Social support (SSRS) (+) – Regular exercise (+) – Normal BMI (+) – Employment (+) – Higher economic income (+) – Survival time over 5 years (+)
Zhou et al. (2021)	China	*N* = 344	F: 100%	Not provided	Gynaecological	– Self disclosure (DDI) (+) – Confrontation (MCMQ) (+) – Perceived social support (MSPSS) (+) – Avoidance (MCMQ) (+) – Acceptance‐resignation (MCMQ) (−) – Higher level of Education (+)
Zhou et al. (2023)	China	312	F: 100%	M: 49.9 SD: 10.2	Gynaecological	– Perceived social support (MSPSS) (+) – Problem‐focused coping (Brief COPE) (+) – Dysfunctional coping (Brief COPE) (+) – Spouse's age (+) – Partners' cancer treatment (+)

Abbreviations: (+), Positive Correlation; (−), Negative Correlation; (U), Curvilinear correlation; (0), No correlation; AAQ‐II, Acceptance and Action Questionnaire‐II; AMT, Autobiographical Memory Test; APGAR, family adaptation, partnership, growth, affection, and resolve; BFS, Benefit Finding Scale; BIS, Body Image Scale; BPNS, Persian version of the 21‐item Basic Psychological Needs Satisfaction Scale; BRCS, Brief Resilient Coping Scale; Brief COPE, Brief Coping Orientation to Problems Experienced Scale; BRS, Brief Resilience Scale; CD‐RISC, Connor–Davidson Resilience Scale; CERQ, Cognitive Emotion Regulation Questionnaire; CFQ, Cognitive Fusion Questionnaire; COPE‐NVI, Coping Orientation to Problems Experienced—New Italian Version; COR‐E, Conservation of Resources Evaluation; CPOTS, Cognitive Processing of trauma scale; CSI, Coping Strategy Indicator; DASS, Depression, Anxiety, and Stress Scale; DCI, Dyadic Coping Inventory; DDI, Distress Disclosure Index; ERQ, Emotion regulation questionnaire; ERRI, Event Related Rumination Inventory; FACT‐B, Functional Assessment of Cancer Therapy‐Breast; FACT‐O/HRQOL, Functional Assessment of Cancer Therapy‐Ovarian/health‐related quality of life; FCR, Fear Of Recurrence; FMI, Freiburg Mindfulness Inventory; GAD‐2, Generalised Anxiety Disorder 2‐item; HADS, Hospital Anxiety and Depression Scale; HS, Hope Scale; I‐CD‐RISC‐10, Italian 10‐item Connor‐Davidson Resilience Scale; IIEF, International Index of Erectile Function; IPC, Internality, Powerful others and Chance; I‐TIPI, Italian Ten Item Personality Inventory (I‐TIPI); JSS, Jenkins Sleep Scale; K‐CCQ, Korean version of the Cancer Coping Questionnaire; KOP‐26, Kwestionariusz Oceny Prężności questionnaire; MAAS, Mindful Attention Awareness Scale; MAS, Marital Adjustment Scale; MAT, Locke–Wallace Marital Adjustment Test; MCMQ, Medical Coping Modes Questionnaire; Mini‐MAC, Mini‐Mental Adjustment to Cancer Scale; MLQ, Meaning in Life Questionnaire; RIS, Multidimensional Rumination in Illness Scale; MSPSS, Multidimensional Scale of Perceived Social Support; PHQ‐12, Patient Health Questionnaire‐12; PHQ‐9, Patient Health Questionnaire; PSS, Posttraumatic Stress Disorder Symptom cale; PSSS, Perceived Social Support Scale; PTSD‐7, Short screening scale for DSM‐IV posttraumatic stress disorder; PTSS, Posttraumatic stress symptoms; QPR, Questionnaire about the Process of Recovery; QSC‐R10, Questionnaire on Stress in Cancer‐Revised Version; RCS, Religious Coping Scale; RS‐14, Resilience Scale; SAI, State Anxiety Inventory; SCNS‐34, 34‐item Supportive Care Needs Survey; SCS, Self‐Compassion Scale; SCSQ, Simplified Coping Style Questionnaire; SCS‐SF, Self‐Compassion Scale‐Sort Form; SPAN‐C, Chinese version of the SPAN (Startle, Physiological Arousal, Anger, And Numbness) Questionnaire; SPBS, Self‐perceived Burden Scale; SSRS, Social Support Rating Scale; SSSQ, Simple self‐coping style questionnaire; SSSS, Sources of Social Support Scale; SWLS, Satisfaction with Life Scale; WAS, World Assumption Scale; WHOQOL, World Health Organisation Quality of Life; WHOQOL‐BREF, World Health Organisation (WHO) Quality of Life‐BREF scale; YSQ‐SF, Young schema questionnaire—short form.

#### 3.4.1 Cognitive and Emotional Coping

This domain was among the more frequently studied and showed robust and consistent associations with PTG across studies. Resilience showed a positive association with PTG across several studies (Choi et al. 2023; Ernst et al. 2023; Gori et al. 2021; Gu et al. 2023; Hlubocky et al. 2022; Li et al. 2020; Longcoy et al. 2023; Michalczyk et al. 2022; Tu et al. 2020; Zhang et al. 2019). Mindfulness was similarly positively correlated with PTG (Aliche 2023; Aliche et al. 2023; Lianchao and Tingting 2020). Coping strategies that involved positive reinterpretation, cognitive reappraisal, problem‐solving, emotional regulation, self‐compassion, and meaning‐making were associated with greater PTG, while passive or avoidant strategies such as acceptance–resignation were negatively associated (Aliche et al. 2023; Karimzadeh et al. 2021; Lisica et al. 2019; Moghadam et al. 2021; Mostarac and Brajković 2022; Peng et al. 2019; Özönder Ünal et al. 2023; Zhang et al. 2021; Zhou et al. 2021). Deliberate rumination—intentional reflection on the traumatic experience—was similarly positively related to PTG (Lianchao and Tingting 2020; Rider Mundey et al. 2019; Song et al. 2021; Szcześniak et al. 2022), in contrast to intrusive rumination. This divergence reinforces theoretical distinctions between types of cognitive processing. These findings support models that frame PTG as a reconstruction of core beliefs and life priorities. Some inconsistencies were noted, however, in the role of emotional intelligence and reappraisal, indicating that contextual and cultural factors may influence these associations (Guil et al. 2022; Rezaee Vessal et al. 2022).

#### 3.4.2 Psychological Symptoms and Burden

Numerous studies demonstrated negative associations between post‐traumatic growth (PTG) and psychological distress, including depression, anxiety, and intrusive rumination. Depression and anxiety were inversely associated with PTG in multiple samples (Aderhold et al. 2019; McErlean et al. 2023; Li et al. 2016; Peng et al. 2019; Thakur et al. 2022). Similarly, intrusive rumination—repetitive, unwanted thoughts typically linked to distress—was found to be negatively associated with PTG and showed a consistent negative correlation with PTG (Gür and Öztürk 2023; Lianchao and Tingting 2020; Szcześniak et al. 2022). However, the evidence was more mixed regarding post‐traumatic stress symptoms. Some studies did not find any association (Honorari et al. 2022); others reported an inverse relationship between PTSD symptoms and PTG (Ernst et al. 2023), and others noted a co‐occurrence, suggesting that PTG and PTSD may arise simultaneously in response to trauma (Baník et al. 2022; Chen et al. 2019; Liu, Zhang, et al. 2020). Another study found that PTG does not correlate significantly with emotional well‐being (Hlubocky et al. 2022). These findings suggest that the relationship may be more nuanced or potentially moderated by other variables such as meaning‐making or coping resources.

#### 3.4.3 Social Support and Interpersonal Variables

Social support emerged as a prominent and consistent correlate of PTG. Multiple studies demonstrated positive associations between PTG and perceived support from family, friends, significant others, and social well‐being (Chen et al. 2019; Hlubocky et al. 2022; Kim and Shin 2022; Kim and Son 2021; Leong Abdullah et al. 2019; Liu, Doege, et al. 2020; Yang and Ha 2019; Romeo et al. 2019; Zhou et al. 2021, 2023). Beyond general support, some studies highlighted the role of dyadic coping and marital satisfaction with mixed findings (Shi et al. 2021; Suo et al. 2021), indicating that relationship quality may be as important as support quantity. The beneficial role of social support extended to peer support and community‐based connections, which also facilitated higher PTG (Jewett et al. 2022). Additionally, social support was found to enhance individuals' perceived and actual levels of personal resources—particularly economic and political ones—which in turn facilitated post‐traumatic growth (Houn et al. 2022).

#### 3.4.4 Treatment‐Related Factors

Associations between PTG and treatment‐related or physical health variables were less frequently explored and revealed more heterogeneous results. Overall, poorer physical health and greater symptom burden—such as fatigue, pain, body image, and functional limitations—were generally associated with lower PTG (Chang et al. 2022; Sheikh‐Wu et al. 2022; Li 2022; Longcoy et al. 2023). Higher unmet physical needs and fear of cancer progression (FCR) were associated with diminished PTG (Nik Jaafar, Hamdan, et al. 2022). Similarly, unresolved symptoms and greater subjective treatment burden were negatively linked to PTG (Zhang et al. 2019; Liu, Doege, et al. 2021). Conversely, better perceived health status, fewer lingering treatment‐related complaints, greater physical recovery, and higher functionality were positively associated with PTG (Daniel et al. 2021; Kim et al. 2021; Liu, Thong, et al. 2021), suggesting that physical recovery may provide a foundation for psychological adaptation. Health care professional (HCP) support and satisfaction also featured as significant associations. Perceptions of adequate time spent by the health care team on information sharing were positively associated with PTG (Evans et al. 2022; Jewett et al. 2022). Similarly, HCP support was positively linked to PTG in brain tumour patients, where it was found to be one of the strongest associations, even more than patient demographics or tumour characteristics (Kim and Shin 2022). Importantly, one study emphasised the role of informed decision‐making: cancer survivors who made an informed choice not to pursue fertility preservation (FP)—due to personal preference or medical guidance—reported higher PTG than those who were unaware or uninformed (Daniel et al. 2021). This suggests that autonomy and clarity in treatment decisions may foster psychological adjustment. These findings underline the importance of not only clinical competence but also relational care in fostering patient PTG.

#### 3.4.5 Spirituality and Religiosity

Spiritual and religious factors were positively associated with PTG across several cultural contexts. Hope, religious beliefs, and spiritual meaning‐making were identified as correlates of higher PTG in cancer patients (Boyacıoğlu et al. 2022; Kim and Son 2021; Leong Abdullah et al. 2019; Moghadam et al. 2021; Ofei et al. 2023; Oh et al. 2021; Yang et al. 2023). In one study, spirituality mediated the relationship between post‐traumatic stress and PTG (Yang et al. 2023). No negative associations between religiosity or spirituality and PTG were reported. Although fewer in number, these studies suggest that spiritual frameworks may provide essential resources for meaning‐making and PTG in the cancer context.

#### 3.4.6 Socioeconomic Status

Socioeconomic status (SES) was identified as a contextual factor that influences PTG, often interacting with psychological and interpersonal variables. Several studies found that lower income was associated with reduced PTG or increased psychological burden. For example, Liu, Doege, et al. (2020) found that breast cancer survivors with lower income were more likely to fall into “resisting” or “struggling” PTG profiles, characterised by lower growth and higher distress. Similarly, Zhang et al. (2020) reported that higher economic income predicted greater PTG, alongside variables such as coping style and social support. In a study of older cancer survivors, Yang and Ha (2019) found that lower SES negatively affected quality of life, while PTG and wisdom were associated with improved well‐being. Additionally, studies by Li et al. (2016) and Longcoy et al. (2023) suggested that income may indirectly influence PTG through its relationship with emotional distress, resilience, and access to resources.

### 3.5 Longitudinal Studies: Predictors and Predicted Variables

Longitudinal studies reviewed were 21 (Table 2). Five of them showed that PTG increased over time (Bourdon et al. 2019; Hamdan et al. 2022; Nik Jaafar et al. 2021; Nik Jaafar, Abd Hamid, et al. 2022; Wang et al. 2023), while one showed that it decreased (Corman et al. 2021) and one that it increased between 6 and 12 months and then again it reached baseline levels (Schwartz et al. 2022). In particular, Hamdan et al. (2022) found that PTG significantly increased over a 5–7 month follow‐up in head and neck cancer (HNC) survivors. However, patients experiencing greater difficulties with social contact (e.g., speaking or interacting due to treatment side effects) and sensory deficits (e.g., taste and smell impairments) reported lower PTG over time. These findings suggest that PTG trajectories may be influenced by cancer‐specific physical complications.

**TABLE 2:** Longitudinal PTG studies.

References	Country of study	*N*	Gender female/male of total %	Population mean age (SD) control	Type of cancer	Time points of PTG measurement	Factors predicting PTG	Factors which PTG predicted
Bourdon et al. (2019)	France	*N* = 293	F: 83.7% M: 15.47 (0.7% missing)	M: 52.5 SD: 9.9	Mixed	Baseline, 6,12,24 months	– Emotional & Positive coping (Brief Cope questionnaire) (+) – Substance Use after 2y (−)	—
Blickle et al. (2024)	Germany	*N* = 1316	F: 51.4% M: 48.6%	M: 67.28 SD: 11.05	Mixed	2 years	– Emotional distress (PHQ‐4) (+) – Fatigue (EORTC QLQ‐FA12) (+) – EORTC – Pain (EORTC QLQ30) (+) – Lower age (+) – Female sex (+) – Receiving chemotherapy (+)	—
Scherer‐Trame et al. (2022)	Germany	*N* = 1906 (100%) Rehabilitants = 934 (49.0%) Non‐rehabilitants = 972 (51.0%)	F: 39.4% M: 60.7%	M: 66.3 at diagnosis and M: 71.3 at follow‐up (±10.4)	Colorectal Cancer	5 years	– Undergoing Rehabilitation (+)	—
Schwartz et al. (2022)	USA	*N* = 430	F: 40.0% M: 60.0%	M: 53 years	Haematological	1, 3, 6, 12 months	– Social support (SPS) (+) – Emotional engagement – (Brief COPE) (+)	—
Senger et al. (2024)	USA	*N* = 123	F: 100%	M: 50.4 SD: 10.3	Breast cancer (BC)	Baseline, 9 and 15 months	– Active coping (Brief‐COPE) (+) – Intrusive thoughts (PTSS) (−)	Physical health‐ related QOL (+)
Liu et al. (2023)	Germany	*N* = 2704	F: 52.1% M: 47.9%	not reported	Mixed	Baseline (2008–2011) Follow up (2018–2019)	—	– Higher **global health status/QOL** (cross‐sectional only) – Higher **role functioning** (cross‐sectional only)
Matsui and Taku (2023)	Japan	N_(T1)_ = 710 N_(T2)_ = 419	F_(t1)_: 50.7% M_(t1)_: 49.3% F_(t2)_: 48.4% M_(t2)_: 51.6%	M: 58.1 SD: 11.9 (t1) M: 58.8 SD: 11.6 (t2)	Mixed	Baseline (2015), Follow up (2016)	– Help seeking behaviour (+) – Receiving psychosocial support (+)	– Help seeking Behaviour (+) *(the relationship appears **bidirectional)***
Mell et al. (2022)	USA	*N* = 154	F: 100%	M: 62.4 SD: 10.4	Mixed	Baseline, 6, 12, 18 months	—	– Cancer Worry Scale (CWS) (+)
Corman et al. (2021)	France	N_(T1)=_ 187 N_(T2)=_ 157 N_(T3)=_ 91	F: 41.9%	M: 52.07 SD: 13.22	Haematological	Baseline, 1 week after HSCT, 5 months after HSCT	– Happiness (SA‐DHS) (+) – Having received Haematopoietic Stem‐Cell Transplantation (−)	—
Nik Jaafar et al. (2021)	Malaysia	*N* = 200	F: 45.5% M: 54.5%	Not reported	Head and Neck Cancer (HNC)	Baseline and follow‐up 5–7 month	– Approach Coping (Active Coping, Planning, Positive reframing, acceptance, emotional support, instrumental support, religious coping) (Brief‐COPE) (+) – Avoidant coping (denial and self‐distraction) (Brief‐COPE) (−)	—
Couderc et al. (2023)	France	*N* = 371	M = 60.8%	56.1 years (SD 9.8) at diagnosis	Lung Cancer (LC)	5 years	– Feeling self‐conscious (EORTC QLQ‐C30) (−) – Suspected neuropathic pain (−)	– Better Mental Quality of life (EORTC QLQ‐C30) (+)
Nik Jaafar, Hamdan, et al. (2022)	Malaysia	175	F: 46.3% M: 53.7%	71% were 41–60 years	Head and Neck Cancer (HNC)	Baseline and follow‐up (5–7 months)	– Hope (DHS) (+) – Perceived spousal support (SSSS) (+) – Anxiety and depression (HADS) (−)	—
Wang et al. (2023)	Taiwan	*N* = 359	F: 100%	M: 47.5 SD: 8.76	Breast cancer (BC)	1 day, 3 months, 6 months, 12 months, and 24 months after surgery	—	– Physical and psychosocial aspects of HRQOL (SF‐36 Health Survey) (U)
Onyedibe et al. (2024)	Germany	*N* = 1316	F: 51.4% M: 26.82%	M: 67.28 SD: 11.05	Mixed	Single time point, 4 years after diagnosis		– Global Health‐Related Quality of Life (EORTC QLQ‐C30) (U)
Philipp et al. (2020)	Germany	*N* = 307	F: 69% M: 31%	M: 59.6 SD: 11.1	Mixed	Baseline and 12‐month follow‐up	—	– Demoralisation (DS): (−)
Rey et al. (2021)	France	*N* = 723	F: 100%	not reported	Breast cancer (BC)	5 years post‐diagnosis		– Physical Activity (VICAN) (cross‐sectional only) (+) – Psychological Distress (−)
Romeo et al. (2020)	Italy	*N* = 147	F: 100%	M: 54.01 SD: 7.84	Breast cancer (BC)	2 years after diagnosis	– Depressive symptoms (HADS): (−)	—
Evans et al. (2022)	France	*N* = 1982	F: 63.8% M: 36.2%	M: 65.7 SD: 11.8	Mixed	2 and 5 years after diagnosis (VICAN)	– Physical activity (+) – Healthier diet (+) – Satisfied with the time spent by health care team on information (+) – Pychological support on diagnosis (+) – Depressive symptoms (HADS) (−)	—
Hamama‐Raz et al. (2019)	Israel	*N* = 198	F: 100%	M: 51.80 SD: 10.85	Breast cancer	3 months after treatment, 1 year, 7 years		– Positive coping strategies (CERQ) (+) (2y) – 7 years after diagnosis, no association was found
Hamdan et al. (2022)	Malaysia	*N* = 200	F: 45.5% M: 54.5%	M: 72.5% aged 41 to 60	Head and Neck (HNC)	Baseline, Follow‐up 5–7 months	– Problems with social contact (EORT‐QLQ‐H&N‐35) (−) – Problems with senses (EORT‐QLQ‐H&N‐35) (−) – Muslim (+) – Buddhist (+)	—
Jie et al. (2020)	China	*N* = 300	(Male/female) F: 17.66% M: 82.33%		Primary Hepatocellular Carcino‐ma (HCC)	At discharge, and 1 month after discharge	– Disclosure of cancer diagnosis (+)	– Quality of life (EORTC QLQ‐C30) (+)

Abbreviations: (+), Positive correlation; (−), Negative correlation; (0), No correlation (U), Curvilinear relationship/convex quadratic relationship; CERQ, The Cognitive Emotion Regulation Questionnaire; COPE, Coping Orientation to Problems Experienced Inventory; CRC, Colorectal Cancer; CWS, Cancer Worry Scale; DHS, Dispositional Hope Scale; EORTC QLQ‐FA12, European Organisation for Research and Treatment of Cancer “Quality of Life Questionnaire”; EORTC QLQ‐C30, European Organisation for Research and Treatment of Cancer; EORTC QLQ‐30, European Organisation for Research and Treatment of Cancer “Core Quality of Life Questionnaire”; EORT‐QLQ‐H&N‐35, European Organisation of Research and Treatment of Cancer's “Quality of Life Questionnaire—Head and Neck 35”; HADS, The Hospital Anxiety and Depression Scale; HCT, Haematopoietic Cell Transplantation; HRQoL, Health‐Related Quality of Life; HSCT, Haematopoietic Stem‐Cell Transplantation; PCL‐C, PTSD Checklist—Civilian Version; PHQ‐4, 4‐item Patient Health Questionnaire; PTGI, Posttraumatic Growth Inventory; PTSS, Post‐Traumatic Stress Symptoms; QLQ‐C30, Quality of Life Questionnaire; QSC‐R10, Questionnaire on Stress in Cancer Patients; SA‐DHS, Subjective Authentic‐Durable Happiness scale; SPS, Social Provisions Scale; SSSS, Sources of Social Support Scale; VICAN, VIe après le CANcer/National survey on French Cancer Survivors.

Assessment measures commonly used, either as complete scales or individual subscales, among the studies were the quality of life questionnaire (QLQ‐C30) (Bourdon et al. 2019), social provisions scale (SPS) (Schwartz et al. 2022), coping orientation to problems experienced (COPE) Inventory (Nik Jaafar et al. 2021), the Brief COPE inventory (Nik Jaafar et al. 2021; Schwartz et al. 2022), dispositional hope scale (DHS), the social support from spouse scale (SSSS) (Nik Jaafar, Abd Hamid, et al. 2022), HADS (Nik Jaafar, Abd Hamid, et al. 2022; Romeo et al. 2020), the EORTC‐QLQ‐H&N‐35 (Hamdan et al. 2022), cancer worry scale (CWS) (Mell et al. 2022), Short Form Health Survey (SF‐36) (Wang et al. 2023), Health Related Quality of Life (EORTC QLQ‐H&N35; EORTC‐QLQ‐C30) (Hamdan et al. 2022; Onyedibe et al. 2024) and the cognitive emotion regulation questionnaire (CERQ) (Hamama‐Raz et al. 2019). Other measures included physical activity levels (Rey et al. 2021) and satisfaction with the time spent by the healthcare team on information (Evans et al. 2022).

#### 3.5.1 Coping Strategies

Multiple studies affirmed the predictive role of coping in fostering PTG. Positive coping strategies such as hope, acceptance, and planning were consistently associated with increases in PTG over time (Nik Jaafar et al. 2021; Senger et al. 2024). Emotional engagement coping (e.g., self‐distraction) similarly predicted enhanced PTG following haematopoietic stem‐cell transplantation (Schwartz et al. 2022). Conversely, avoidant coping strategies (e.g., denial) were linked to decreased PTG levels (Nik Jaafar et al. 2021), and substance use was similarly associated with lower PTG at follow‐up in both melanoma and breast cancer populations (Bourdon et al. 2019). In turn, PTG itself emerged as a positive predictor of adaptive coping strategies, suggesting a reciprocal relationship. For instance, PTG predicted greater use of positive cognitive emotion regulation strategies at 2‐year follow‐up among breast cancer patients (Hamama‐Raz et al. 2019).

#### 3.5.2 Emotional and Psychological Predictors

Several studies investigated the influence of psychological factors on PTG. A study showed that the magnitude and trajectory of change differed depending on levels of depressive symptoms, with higher depression generally limiting PTG but not eliminating it (Bourdon et al. 2019). High levels of depressive symptoms at diagnosis, when resolved over time, were predictive of increased PTG (Romeo et al. 2020), whereas persistent anxiety symptoms were negatively associated with PTG (Nik Jaafar, Abd Hamid, et al. 2022). PTG itself was found to influence psychological outcomes with mixed results. A study revealed a curvilinear (U‐shaped) relationship between PTG and mental health, with both high and low PTG levels being associated with better outcomes than moderate PTG levels (Wang et al. 2023). Interestingly, PTG was linked to increased cancer worry in gynecologic cancer survivors (Mell et al. 2022), suggesting that PTG may, under certain circumstances, co‐occur with elevated health‐related concerns.

#### 3.5.3 Social Support and Relational Factors

Social support emerged as a positive predictor of PTG. For example, perceived spousal support was strongly associated with PTG trajectories among head and neck cancer patients (Nik Jaafar, Abd Hamid, et al. 2022). Similarly, Disclosure of diagnosis from family and friends is beneficial for patients in reducing PTSS and improving PTG, as it allows open communication and informational support (Jie et al. 2020). Notably, one study reported that higher pre‐transplant social support, along with emotional coping, predicted increased PTG at multiple follow‐up points (Schwartz et al. 2022). In turn, Problems with social contact predicted lower PTG levels over time (Hamdan et al. 2022). Furthermore, higher PTG—especially in the domains of “Appreciation of Life” and “New Possibilities”—predicted greater use of psychosocial support services over time, suggesting a reinforcing cycle between psychosocial engagement and PTG (Matsui and Taku 2023). Furthermore, higher relatedness to others (PTG) predicted lower demoralisation (Philipp et al. 2020).

#### 3.5.4 Medical and Health‐Related Factors

Several medical variables also influenced PTG longitudinally. Physical activity predicted greater PTG (Evans et al. 2022) and at 5‐year follow‐up in breast cancer survivors (Rey et al. 2021), while in turn, PTG predicted improved physical and mental health outcomes (Wang et al. 2023). Additionally, satisfaction with healthcare information (Blickle et al. 2024) and psychological support at diagnosis were associated with higher PTG levels, suggesting that system‐level care factors can influence psychological outcomes (Evans et al. 2022). Fatigue and pain emerged as non‐linear predictors of PTG. Additionally, among HNC patients, persistent problems with the senses (e.g., dysgeusia, anosmia) and social contact difficulties significantly predicted lower PTG over time, underlying that lingering physical complications can interfere with trauma processing and meaning‐making (Hamdan et al. 2022). In breast cancer patients, greater PTG at 9 months predicted better physical health‐related quality of life (HRQoL) at 15 months, particularly among those practising active coping (Senger et al. 2024). Liu et al. (2023) found that higher PTG at the start tended to do better in HRQoL over time, especially in role functioning and global health—though not all improvements were statistically significant. However, one study revealed a curvilinear (U‐shaped) relationship between PTG and Health Related Quality of Life (HRQoL) (Wang et al. 2023). Another found a convex quadratic relationship between PTG and HRQoL not moderated by fatigue, emphasising the nuanced nature of these associations (Onyedibe et al. 2024).

### 3.6 Interventions

Thirteen interventional studies were found (Table 3), of which 11 were randomised control trials (RCTs) (Akechi et al. 2023; Han et al. 2021; Holtmaat et al. 2019; Kissane et al. 2023; Lleras de Frutos et al. 2020; Ochoa‐Arnedo et al. 2021; Tu et al. 2021; Wang, Li, et al. 2022; Wang, Lin, et al. 2022; Yun et al. 2020; Zhu et al. 2022), and two were pilot (Chu et al. 2022; Kissane et al. 2019). The studies included interventions based on positive psychotherapy (Lleras de Frutos et al. 2020; Ochoa‐Arnedo et al. 2021; Tu et al. 2021), interventions focusing on meaning‐making (Holtmaat et al. 2020; Kissane et al. 2019, 2023), problem‐solving therapy and behavioural activation (Akechi et al. 2023), mindfulness‐based stress‐reduction (Zhu et al. 2022), nurse‐led supportive‐expressive group therapy (Wang, Li, et al. 2022), Naikan and Morita therapies (Han et al. 2021), health coaching (Yun et al. 2020), culturally sensitive educational lectures and peer mentor support (Chu et al. 2022) and interventions based on the biopsychosocial model focusing on the continuity of care (Wang, Lin, et al. 2022). Six studies showed significant changes to PTG in the intervention group compared with the control group (Han et al. 2021; Kissane et al. 2023; Tu et al. 2021; Wang, Li, et al. 2022; Yun et al. 2020; Zhu et al. 2022), while five studies revealed significant improvement in PTG post‐intervention, compared to baseline (Kissane et al. 2023; Lleras de Frutos et al. 2020; Wang, Li, et al. 2022; Yun et al. 2020; Zhu et al. 2022).

**TABLE 3:** Interventional studies on PTG.

References	Country of study	Study type	N	Gender female/male of total (%)	Population mean age (M), standard deviation (SD)	Type of cancer	Within	Between
Chu et al. (2022)	USA	8‐week pilot culturally sensitive group support intervention vs. routine care	*N* = 195 Intervention (*n* = 86) Routine Care (*n* = 109)	F: 100%	M: 55.31; SD: 8.47 (Intervention)/ 58.43; SD: 8.92 (Routine Care)	Breast cancer	(+) Only the intervention group demonstrated significant PTG improvement across time.	(+) The intervention group demonstrated significantly higher PTG scores in comparison to the control group at follow‐up.
Akechi et al. (2023)	Japan	Smartphone problem‐solving therapy and behavioural activation vs. control	*N* = 444 Intervention (*n* = 223) Control (*n* = 224)	Not reported	M: 43.9 SD: 4.57 (intervention)/ M: 44.0/SD: 4.49 (control)	Breast cancer	0 No significant PTG improvement observed over time in either group.	0 No significant difference in PTG scores between the smartphone psychotherapy group and the waitlist control.
Lleras de Frutos et al. (2020)	Spain	Positive psychotherapy for cancer survivors (PPC) vs. Online group positive psychotherapy for cancer survivors (OPPC)	*N* = 269 PPC (*n* = 145) OPPC (*n* = 124)	F: 100%	M: 52.17 SD: 8.36 (PPC)/ M: 47.34 SD: 8.05 (OPPC)	Mixed	(+) Both groups reported increased PTG across time.	0 There is no significant difference between the groups, and no control group to compare them to.
Zhu et al. (2022)	China	Mindfulness‐Based Stress Reduction Therapy vs. routine care	*N* = 101 Intervention (*n* = 50) Routine Care (*n* = 51)	F: 100%	M: 48.88 SD: 8.017	Breast cancer	(+) Both groups showed significant improvement in PTG scores from baseline to post‐intervention.	(+) The intervention group exhibited significantly greater PTG improvements compared to the routine care group across time.
Wang, Lin, et al. (2022)	China	Nurse‐led support intervention vs. Control	*N* = 168 Intervention (*n* = 84) Control (*n* = 84)	F: 100%		Breast cancer	(+) Significant increase in PTG over time in both groups; the intervention group showed significant within‐group gains across all PTG domains except spiritual growth.	(+) One month post‐intervention, the intervention group showed significantly higher total PTG and improvements in 4 PTG domains (except spiritual change) compared to the control group.
Han et al. (2021)	China	Naikan and Morita Therapies vs. Control	*N* = 130 Intervention (*n* = 65) Control (*n* = 65)	F: 66.15% M: 33.85%	M = 58.04 SD = 14.21	Mixed	(+) The intervention group showed significant improvement from pre‐ to post‐treatment in total PTG and all five PTGI domains. No significant change was observed in the control group.	(+) At post‐treatment, the intervention group had significantly higher PTG scores overall and on each PTGI domain compared to the control group.
Tu et al. (2021)	China	Positive psychological intervention vs. Control	*N* = 100 Intervention (*n* = 50) Control (*n* = 50)	F: 42% M: 58% (intervention)/ F: 46% M: 54% (control)	M: 56.41 SD: ±8.14 (intervention)/ M: 57.32 SD: ±7.89 (Control)	Lung cancer	(+) Both groups showed increased PTG scores after the intervention, with the intervention group showing greater improvement.	(+) The intervention had significantly higher PTGI scores in comparison to the control group.
Ochoa‐Arnedo et al. (2021)	Spain	Cognitive behavioural stress management vs. (CBSM) vs. Positive psychotherapy in cancer (PPC)	*N* = 140 CBSM (*n* = 73) PPC (*n* = 67)	F: 100%	M: 49.68 SD: 10.18 (CBSM)/ M: 50.81 SD: 9.49 (PPC)	Mixed	0 Neither the PPC nor the CBSM group showed a statistically significant increase in PTG scores from pre‐ to post‐intervention or follow‐ups.	0 PTG scores were slightly higher in the PPC than the CBSM group over time, but differences were not statistically significant.
Wang, Lin, et al. (2022)	China	Extended care intervention based on the biopsychosocial medicine model vs. Control (usual extended care)	*N* = 152 Intervention (*n* = 76) Control (*n* = 76)	F: 40.78% M: 59.21% (intervention)/ F: 42.1 M: 57.89% (control group)	M: 52.84 SD: ±5.13 (intervention)/ M: 52.77 SD: 5.09 (control group)	Abnormal tumour markers	(+) Both groups showed significant increases in total PTG and all five subscales over time, but greater improvements were observed in the intervention group.	(+) The intervention group had significantly higher total PTG and higher scores in four subscales than the control group. No between‐group difference in spiritual change.
Yun et al. (2020)	Republic of Korea	Health coaching + web group vs. Web‐only group vs. Control	*N* = 394 Health coaching + web group (*n* = 135) Web‐only group (*n* = 125) Control (*n* = 134)	F: 62.2% M: 37.8% (Health coaching + web group)/ F: 60.8 M: 39.2 (Web‐only group)/ F: 60.4 M: 39.6 (Control)	M: 52.69 SD: 10.52 (Health coaching + web group)/ M: 54.37 SD: 11.04 (Web‐only group)/ M: 54.39 SD: 11.02 (Control)	Mixed	(+) Significant increase in PTGI scores at 12 months observed only in the health coaching + web group: no significant change in web‐only or control groups.	(+) At 12 months, the Health coaching + web group showed a significant improvement in PTGI compared to the control group. No significant difference in PTGI was found between the web‐only and control groups.
Holtmaat et al. (2020)	Netherlands	Meaning‐centered group psychotherapy for cancer survivors (MCGP) vs. Supportive group psychotherapy (SGP) vs. Care as usual (CAU)	*N* = 170 MCGP (*n* = 57) SGP (*n* = 56) CAU (*n* = 57)	F: 70% (MCGP)/ F: 88% (SGP)/ F: 90% (CAU)	M: 59 (MCGP)/ M: 56 (SGP)/ M: 57 (CAU)	Mixed	(+) Significant increase in PTGI (personal growth subscale) scores in the MCGP group across the 2‐year follow‐up. No significant change in the SGP or CAU groups.	(+) At 2‐year follow‐up, MCGP reported significantly greater personal growth compared to SGP.
Kissane et al. (2019)	Australia	Meaning and Purpose Therapy (MaP) vs. Wait‐list control	*N* = 57 MaP (*n* = 30) wait‐list control (*n* = 27)	M: 53%	M: 65 SD: 12.9	Mixed	(+) PTG scores increased in the MaP group across several domains.	(+) In the full intervention group (including crossover), MaP showed moderate effect sizes vs. control, but initial between‐group differences were not statistically significant.
Kissane et al. (2023)	Australia	Meaning and Purpose Therapy (MaP) vs. Control	*N* = 107 MaP (*n* = 55) Control (*n* = 52)	F: 72.7% M: 27.3% (MaP)/ F: 78.7% M: 21.3% (control)	M: 60.4 SD: 11.0 (MaP)/ M: 63.2 SD: 11.8 (control)	Mixed	(+) The MaP group showed significant increases in PTGI scores post‐intervention, which remained moderate at follow‐up. The control group showed no significant change over time.	(+) The MaP group showed significantly higher PTG than the control group post‐intervention, but not at follow‐up.

*Note*: (−), Negative Association, statistically significant; (+), Positive Association, statistically significant; —, No PTG correlation reported in the results of the study, despite its inclusion in the methodology; 0, No significant correlation, reported by the study's result; PTGI, posttraumatic growth inventory.

## 4 Discussion

This review aimed to assess recent studies on PTG among cancer patients and survivors over the past 5 years to identify psychological factors associated with PTG and interventions that foster PTG. The findings indicate substantial inclusion of PTG measures in recent research across numerous studies.

A total of 109 studies focusing on PTG in cancer populations were identified in the past 5 years. The majority of participants in these studies were female, with a significant portion of research specifically examining PTG in breast and gynaecological cancer patients. The number of studies on breast cancer may be explained by the fact, as recent data reveals, that there has been an escalating trend in breast cancer cases since 2018, with projections indicating a continued rise in the coming years (Cao et al. 2021; Siegel et al. 2021; Soerjomataram and Bray 2021). Notably, a substantial proportion of these studies were conducted in China, possibly reflecting a significant burden of cancer in this region, with China contributing significantly to global cancer statistics, accounting for 24% of newly diagnosed cases and 30% of the cancer‐related deaths worldwide in 2020 (Cao et al. 2021). While this may partially explain the volume of research, it is also possible that growing academic interest and institutional support for psychosocial oncology in the region have contributed to this trend. The predominant study design observed was cross‐sectional, limiting the ability to draw conclusions about the developmental trajectory of PTG and to draw causal inferences of the relationship between the constructs. Fewer studies utilised longitudinal designs, and discrepancies in reporting results impede conclusive interpretations regarding changes in PTG over time (i.e., Couderc et al. 2023; Matsui and Taku 2023; Mell et al. 2022).

Across these studies, PTG was found to reflect a dynamic interplay of cognitive, emotional, social, and health‐related factors (Choi et al. 2023; Kim and Shin 2022; Liu, Doege, et al. 2021). On one hand, positive psychological constructs were robustly linked to greater PTG: for example, resilience and adaptive coping strategies (such as positive reappraisal, deliberate rumination, and meaning‐making) showed strong positive associations with PTG (Aliche et al. 2023; Gür and Öztürk 2023; Gu et al. 2023; Zhou et al. 2023). Mindfulness and a strong sense of meaning in life were also consistently correlated with higher PTG (Aliche 2023; Moghadam et al. 2021; Mostarac and Brajković 2022). By contrast, emotional distress and maladaptive processes tended to undermine PTG: depression, anxiety, and intrusive (unintentional) rumination were generally inversely related to PTG (Aderhold et al. 2019; Gür and Öztürk 2023), although studies indicate that PTG can co‐exist with post‐traumatic stress—they are not simply opposite ends of a spectrum (Shand et al. 2015). Social context likewise played a clear role. Perceived social support—whether from family, peers, or healthcare providers—emerged as a facilitator of PTG (Cordova et al. 2001; Manne et al. 2004; Jewett et al. 2022; Roohi et al. 2020; Mehraban et al. 2022). This finding was also supported by longitudinal studies that showed social support and social integration work as predictors of PTG (Nik Jaafar, Hamdan, et al. 2022; Schwartz et al. 2022). Greater symptom burden and poor physical health typically constrained PTG (Hamdan et al. 2022; Jewett et al. 2022; Kim and Shin 2022; Leong Abdullah et al. 2019). However, disease‐related variables (e.g., cancer type, stage, treatment intensity) showed inconsistent or minimal associations with PTG, suggesting that the capacity for PTG is not confined to particular diagnoses or clinical profiles (Casellas‐Grau et al. 2017). Longitudinal evidence, while limited, indicates that cognitive processing of the cancer experience is a crucial predictor of who develops PTG. Survivors who actively engage in making sense of their illness—for instance through deliberate rumination, positive reframing of the trauma, or finding meaning in the experience—tend to show greater subsequent PTG (Blickle et al. 2024). This highlights that it is the psychological and social context of the survivorship experience—rather than objective disease severity—that most strongly correlates with PTG.

*Resilience* emerges as a particularly central construct—a factor often associated with overcoming adversities. Resilience is characterised by the capacity to sustain stable psychological well‐being amidst prolonged stress, such as enduring chronic illnesses like cancer (Casellas‐Grau et al. 2017), which has been closely linked with the use of coping strategies. These relationships may better be explained by their interconnectedness and potential facilitation of each other. Patients with higher resilience demonstrated more significant employment of adaptive coping strategies, such as acceptance and positive re‐evaluation, correlating with a better quality of life perception (Macía et al. 2021). Therefore, *adaptive coping*, notably problem‐focused coping, self‐efficacy, and acceptance, was positively associated with resilience (Macía et al. 2021). Moreover, resilience has been found to act as a moderator, enhancing the effectiveness of adaptive coping strategies while mitigating potential negative effects associated with maladaptive coping (Smith et al. 2016).

In this context, the role of rumination exemplifies PTG's psychological complexity. *Deliberate (reflective) rumination‐intentional*, constructive processing of the cancer experience‐was consistently associated with higher PTG (Jim and Jacobsen 2008). In contrast, *intrusive rumination‐*automatic, repetitive negative thoughts‐was negatively associated with PTG (Gür and Öztürk 2023; Lianchao and Tingting 2020). This divergence supports cognitive‐processing models: purposeful meaning‐searching can help survivors reframe adversity and reconstruct beliefs, whereas unbidden negative rumination tends to prolong distress. According to the meaning‐making model (Almeida et al. 2022), when people experience something stressful, they attempt to appraise its situational meaning, considering a variety of factors, such as the aetiology, threat level, controllability, and implications for their future. When there is a discrepancy between the situational meaning and the more global meaning of the stressful event in one's life (e.g., the individual's sense of control, coherence, justice, predictability, and sense of purpose), distress is created, resulting in meaning‐making efforts to minimise such discrepancy, that is, by altering the meaning of the stressful event, their global life meaning, or both. Initially, rumination may be intrusive; however, it gradually transitions to a more intentional form, reflecting the activation of cognitive processing geared towards reconstructing fundamental beliefs shattered by the trauma (Janoff‐Bulman 2004; Tedeschi and Calhoun 2004; Nolen‐Hoeksema et al. 2008).

*Mindfulness* is another often cited factor associated with PTG. Mindfulness refers to balancing one's focus in a particular way: purposefully, in the present moment, and non‐judgmentally (Shapiro et al. 2006). Mindfulness practices may foster PTG by promoting positive reappraisal of experiences and enhancing self‐compassion (Aliche 2023). Just like deliberate rumination, this thinking process also appears to promote PTG.

The evidence on emotional distress more broadly was mixed. As noted, higher depressive or anxiety symptoms generally correlated with lower PTG (Nik Jaafar, Abd Hamid, et al. 2022). However, longitudinal analyses revealed that these relationships are not straightforward. For instance, very high depression at diagnosis was found to limit PTG but not eliminate it (Bourdon et al. 2019), and patients whose depression remitted over time often later showed increases in PTG (Romeo et al. 2020). These findings suggest that some distress can co‐occur with, and even precede, growth—PTG may develop through the struggle with negative emotions, rather than only in their absence. In fact, some studies reported U‐shaped or curvilinear patterns: moderate PTG was associated with worse outcomes than either low or very high PTG levels (Wang et al. 2023). Overall, our review indicates that simple linear models of PTG and distress are inadequate. However, the evidence suggests that some degree of emotional challenge is a precursor to PTG, reinforcing the idea that “struggling with” the cancer experience is what catalyses PTG.

In our review, we identified only six intervention studies that effectively facilitated significant changes in PTG compared to control groups, with most demonstrating notable improvement post‐intervention. These studies utilised diverse therapeutic approaches, including Naikan and Morita therapies (Han et al. 2021), positive psychotherapy (Tu et al. 2021), supportive‐expressive group therapy (Wang, Li, et al. 2022), mindfulness‐based stress reduction (Zhu et al. 2022), health coaching (Yun et al. 2020), and existential psychotherapy (Kissane et al. 2023). Notably, the interventions that fostered PTG commonly integrated elements positively associated with PTG. Naikan, Morita, mindfulness, and positive therapies incorporated components of acceptance, self‐compassion, deliberate rumination, and mindfulness. Supportive‐expressive group therapy incorporated social support, while existential psychotherapy focused on enhancing meaning in life and prospects. Consequently, these therapies facilitate PTG through mechanisms aligned with factors that correlate positively with PTG. While intervention outcomes are positive overall, they must be interpreted with caution. Still, the consistent finding that PTG can be enhanced through targeted psychosocial support has important implications for survivorship care, as discussed below.

Despite the diversity of cancers, cultures, and designs, certain core correlates of PTG emerged repeatedly in studies spanning Asia, Europe, and North America. Resilience, social support, deliberate coping, and cognitive processing (especially positive reinterpretation and meaning‐making), mindfulness, spirituality, and higher socioeconomic resources consistently predicted higher PTG (Boyacıoğlu et al. 2022; Kim and Son 2021). Income and other socioeconomic factors also appeared repeatedly as background modulator; studies found that lower income or unaddressed financial stress tended to reduce PTG, presumably by limiting access to support and resources (Yang and Ha 2019; Zhang et al. 2020). By systematically mapping 109 recent studies, our review clarifies that these psychosocial variables are robust PTG predictors across cancer types. In sum, cognitive–emotional resources and strong social/contextual supports are reliably associated with PTG in cancer survivorship, a pattern that holds across diverse patient groups.

The findings of this review offer several insights regarding established theories of PTG. First, they largely support and refine the foundational model of PTG proposed by Tedeschi and Calhoun. According to Tedeschi and Calhoun's framework, PTG arises from the struggle to rebuild one's assumptive world after trauma, facilitated by cognitive processing and social support, and results in growth across distinct life domains (relationships, new possibilities, personal strength, appreciation of life, and spirituality). In our review, we observed clear evidence for these core propositions. For example, greater resilience and meaning of life showed positive correlation with PTG. Moreover, the prominent role of social support and disclosure in facilitating PTG aligns with the theoretical notion that discussing one's trauma with supportive others helps to construct meaning and solidify PTG (Shand et al. 2015). Adaptive coping is one of the strongest correlations of PTG (Blickle et al. 2024), which is also consistent with the model's emphasis on cognitive processing. Tedeschi and Calhoun highlighted that deliberate rumination (thoughtfully reflecting on the trauma and its implications) is critical for PTG, and our review finds that indeed survivors who engage in such reflective processing report more PTG. Our review appears to be in line with the PTG theoretical model that suggests individuals who actively grapple with the existential challenges posed by cancer, re‐evaluate their priorities, and receive empathetic support are more likely to experience transformational positive change.

At the same time, our findings suggest some refinements to existing theories may be warranted to fully capture PTG in cancer contexts. One consideration is the role of ongoing health threats and behavioural changes, which classical PTG models have not emphasised. PTG was a predictor of lifestyle changes (Menger et al. 2021; Rey et al. 2021). These behaviour changes could be viewed as an adaptive response specific to life‐threatening illness. Theoretical models of PTG might be extended to acknowledge such illness‐specific growth manifestations. Additionally, the reviewed evidence calls into question the idea that more severe trauma yields more PTG. Tedeschi and Calhoun had proposed that highly “seismic” events are more likely to shatter core beliefs and thus enable greater growth, provided the person copes constructively. However, in cancer research, objective indicators of illness severity (e.g., stage of disease, invasive treatments) have not shown a clear linear relationship with PTG (Casellas‐Grau et al. 2017). Many patients with early‐stage or less aggressive cancers still report substantial growth, whereas some with very advanced disease do not—suggesting that subjective appraisal and coping processes are more pivotal than the trauma magnitude per se. This nuance aligns with the cognitive adaptation theory (Taylor 1983), which posits that it is an individual's perception and cognitive processing of a threatening event, rather than the event's objective features, that drive adjustment outcomes (Czajkowska 2020). Cognitive adaptation theory emphasises that people strive to restore meaning, mastery, and a positive self‐image after trauma, often through “positive illusions” like optimism and benefit‐finding. These self‐protective cognitive biases may account for PTG in some individuals (Grace et al. 2015) with a study showing that PTG can increase between 6‐ and 12‐months post‐trauma but then decline back to baseline levels thereafter (Schwartz et al. 2022). This suggests that what is reported as growth may, in some cases, represent a temporary coping mechanism rather than a lasting psychological change. Longitudinal evidence is needed to clarify whether PTG persists over time or diminishes as the initial threat decreases. Moreover, the curvilinear relationship observed in another study (Wang et al. 2023)—where moderate levels of PTG were linked to the lowest quality of life—suggests that for some individuals, the transformation associated with PTG may not fully materialise. This may result from the ongoing emotional burden of cognitive processing or unresolved meaning‐making (Wang et al. 2023). Such findings underscore the importance of fostering and supporting deeper processes of growth, rather than assuming that all reports of PTG reflect genuine or beneficial change. Our review showed that cancer survivors who engage in positive adaptive strategies and have a supportive environment are precisely those who exhibit higher PTG. Furthermore, the finding that PTG correlates positively (albeit mildly) with post‐traumatic stress symptoms in cancer shows that PTG and distress are not mutually exclusive and can progress in parallel as independent outcomes of trauma processing. In sum, the reviewed evidence reinforces existing theoretical models—particularly by underlining the importance of adaptive coping and social support as drivers of PTG—while also highlighting unique considerations (like health behaviour change and the weak link to trauma severity) that theories of PTG may need to accommodate when applied to serious illness contexts.

However, because most studies were cross‐sectional, it remains difficult to discern to what extent PTG leads to improved outcomes versus simply co‐occurring with them. Longitudinal research is needed to test theoretical models more rigorously. Overall, the converging evidence from this scoping review strengthens confidence in the major PTG theories by demonstrating their applicability in the cancer context, while also suggesting areas where these models might be extended (incorporating ongoing health threats, differentiating genuine versus illusory growth, fostering PTG) to more fully explain the experience of cancer survivors.

The conclusions of our scoping review both build upon and extend the findings of previous reviews of PTG in cancer populations. We observed similar findings on PTG, reaffirming previous results (Casellas‐Grau et al. 2017; Menger et al. 2021). In terms of scope and methodology, our scoping review differs from most prior reviews, which were typically focused on specific subsets of studies. Our review extends prior work by discussing these outcomes in the context of theoretical models and overall survivorship care. Furthermore, by adopting a scoping methodology, we intentionally cast a wider net across study designs and cancer populations.

While this scoping review offers valuable insights into the overarching concept of PTG, it is essential to acknowledge certain limitations inherent in the study design. Notably, the examination primarily focused on PTG as a holistic construct, without delving into its individual subscales or components. While this approach allowed for a comprehensive understanding of the broader concept, it may have overlooked nuances and variations within specific dimensions of PTG. The review was limited to English‐language publications from 2018 to 2024. As a result, important findings in other languages or older studies may be underrepresented. A predominance of cross‐sectional designs (74 of 109 studies) restricts causal inference. Few investigations tracked PTG over multiple time points, making it hard to determine whether factors like coping genuinely cause later growth. Future work should address these gaps. More longitudinal and intervention studies are needed to trace PTG trajectories and uncover causal processes. Our review found only a handful of trials, which have shown modest improvements using techniques like mindfulness, supportive‐expressive group therapy, and meaning‐centered approaches. These promising programs typically incorporate the factors linked to PTG: for instance, they foster acceptance, deliberate reflection on life priorities, self‐compassion, and social connection. Future RCTs should build on these insights by explicitly targeting and measuring PTG (using the PTGI or similar) and by enrolling larger, more diverse samples. Ultimately, interventions that cultivate mindful present focus, purposeful coping, and meaning making may prove most effective at promoting PTG. Finally, future research could benefit from an exploration of the subscales comprising post‐growth, thereby providing a better understanding of its underlying mechanisms and implications.

## Author Contributions

**Anna Nisiraiou:** Conceptualization, Investigation, Writing – Original Draft Preparation, Writing – Review & Editing, Visualization. **Antonios Bozas:** Conceptualization, Investigation, Writing – Original Draft Kyrou Preparation, Writing – Review & Editing, Software. **Dimitrios Kyrou:** Conceptualization, Investigation, Writing – Original Draft Preparation, Software, Visualization. **Konstantina Stavrogianni:** Writing – Original Draft Preparation, Software, Visualization. **Maria Vasilopoulou:** Writing – Original Draft Preparation, Writing – Review & Editing. **Georgios ‐ Marios Kalomoiris:** Writing – Original Draft Preparation. **Natalia Tsiftsa:** Writing – Original Draft Preparation. **Katerina Nikitara:** Writing – Review & Editing. **George Koulierakis:** Methodology, Writing – Review & Editing, Validation. **Christina Karamanidou:** Conceptualization (lead), Writing – Review & Editing, Methodology (lead), supervision, Validation.

## Conflicts of Interest

The authors declare no conflicts of interest.

## Data Availability

The data that support the findings of this study are available from the corresponding author upon reasonable request.
